# Moderate- to high-intensity statins for secondary prevention in patients with type 2 diabetes mellitus on dialysis after acute myocardial infarction

**DOI:** 10.1186/s13098-017-0272-7

**Published:** 2017-09-19

**Authors:** Yan-Rong Li, Sung-Sheng Tsai, Yu-Sheng Lin, Chang-Min Chung, Szu-Tah Chen, Jui-Hung Sun, Miaw-Jene Liou, Tien-Hsing Chen

**Affiliations:** 1 Division of Endocrinology and Metabolism, Department of Internal Medicine, Chang Gung Memorial Hospital, Linkou, Taiwan; 2 Division of Cardiology, Chang Gung Memorial Hospital, Chiayi, Taiwan; 3 School of Traditional Chinese Medicine, College of Medicine, Chang Gung University, Taoyuan, Taiwan; 4 Division of Cardiology, Department of Internal Medicine, Chang Gung Memorial Hospital, No. 222, Maijin Road, Keelung, Taiwan; 5 Chang Gung University College of Medicine, Taoyuan, Taiwan

**Keywords:** Acute myocardial infarction, Dialysis, Mortality, Statins, Secondary prevention, Type 2 diabetes mellitus

## Abstract

**Background:**

Evidences support the benefits of moderate- to high-intensity statins for patients with acute myocardial infarction (AMI) except for those with type 2 diabetes mellitus (T2DM) on dialysis after AMI. This study was aimed to investigate the safety and efficacy of secondary prevention of cardiovascular diseases using moderate- to high-intensity statins in T2DM patients on dialysis after AMI.

**Methods:**

A simulated prospective cohort study was conducted between January 1st, 2001 and December 31st, 2013 utilizing data from the Taiwan National Health Insurance Research Database. A total of 882 patients with T2DM on dialysis after AMI were selected as the study cohort. Cardiovascular efficacy and safety of moderate- to high-intensity statins were evaluated by comparing outcomes of 441 subjects receiving statins after AMI to 441 matched subjects not receiving statins after AMI. The primary composite outcome included cardiovascular death, non-fatal myocardial infarction and non-fatal ischemic stroke.

**Results:**

The Kaplan–Meier event rate for the primary composite outcomes at 8 years was 30.2% (133 patients) in the statin group compared with 25.2% (111 patients) in the non-statin group (hazard ratio [HR], .98; 95% confidence interval [CI] .76–1.27). Significantly lower risks of non-fatal ischemic stroke (HR, .58; 95% CI .35–.98) and all-cause mortality (HR, .70; 95% CI .59–.84) were found in the statin group.

**Conclusions:**

In T2DM patients on dialysis after AMI, the use of moderate- to high-intensity statins has neutral effects on composite cardiovascular events but may reduce risks of non-fatal ischemic stroke and all-cause mortality.

**Electronic supplementary material:**

The online version of this article (doi:10.1186/s13098-017-0272-7) contains supplementary material, which is available to authorized users.

## Background

Type 2 diabetes mellitus (T2DM) is considered as an equivalent of coronary heart disease [1], with a twofold higher mortality rate than those without T2DM [2]. Among patients with T2DM, chronic kidney disease (CKD) is a predominant independent risk factor for cardiovascular disease (CVD) and death [3]. The risk for premature CVD is increased by 25–30% in early-stage CKD [4], and 30- to 50-fold higher in end-stage renal disease (ESRD) than in people with normal renal function [5]. The complications of CVD are a leading cause of deaths in patients with T2DM and ESRD, accounting for about 50% of all-cause mortality [6–10].

Numerous trials of low-density lipoprotein cholesterol (LDL-C)-lowering treatment with 3-hydroxy-3-methylglutaryl coenzyme A (HMG-CoA) reductase inhibitors (statins) have shown benefits of primary or secondary prevention for CVD in patients not receiving dialysis [11–13]. Current evidence indicates that moderate- to high-intensity statins should be initiated if patients not receiving dialysis have clinical atherosclerotic cardiovascular disease (ASCVD) such as acute coronary syndromes [11]. Nevertheless, for patients on dialysis, guidelines of the 2013 kidney disease: improving global outcomes (KDIGO) and the National Kidney Foundation Kidney Disease Outcomes Quality Initiative (KDOQI) work groups advise that statins treatment should not be administered routinely [14, 15], because the results of major trials such as the Die Deutsche Diabetes Dialyse (4-D), a study to evaluate the use of rosuvastatin in subjects on regular hemodialysis: an assessment of survival and cardiovascular events (AURORA), and the study of heart and renal protection (SHARP), revealed no definite clinical benefits with statins treatment in patients on dialysis [7, 16, 17]. However, one caveat to be considered in the implementation of these guidelines is that patients with recent acute coronary syndrome who may be considered for statins treatment were typically excluded from previous clinical trials [15]. Moreover, secondary outcomes in the 4-D study showed significant 18% reductions in the rate of combined cardiac events (hazard ratio [HR] .82; 95% confidence interval [CI] .68–.99). Post-hoc analysis of the AURORA trial found that rosuvastatin significantly reduced rates of cardiac events (including cardiac death and non-fatal myocardial infarction) by 32% (HR .68; 95% CI .51–.90) in diabetic patients [18].

Although secondary outcomes in the 4-D trial and post hoc analysis of the AURORA trial suggested possible benefits of statins among diabetic patients on dialysis, the major limitation of interpretation is that findings of secondary outcomes and subgroup analysis, respectively, were not predefined [19]. In addition, in the AURORA trial, evaluation of the safety of statins treatment revealed a higher incidence of hemorrhagic stroke in the rosuvastatin group, although the number of events was small (12 vs. 2, respectively; HR 5.21; 95% CI 1.17–23.27) [16]. As a result, the clinical benefits of statins in patients with T2DM on dialysis after acute myocardial infarction (AMI) are still uncertain. Therefore, this study was aimed to evaluate the efficacy and safety of moderate- to high-intensity statins in patients with T2DM on dialysis after AMI.

## Methods

### Data source

The National Health Insurance (NHI) program covers more than 99% of 23 million people in Taiwan. All submitted standardized information and data of healthcare services are prospectively recorded by the NHI research database (NHIRD). Diagnoses are registered using the international classification of diseases, ninth revision, clinical modification (ICD-9-CM) codes. The NHI Bureau routinely and comprehensively performs validation of accurate records of beneficiaries, including ambulatory visits, inpatient care, disease diagnosis codes and medication prescriptions from the NHIRD data [20–23]. The nationwide NHIRD is an important source of data and contributory for many large population-based studies [24]. The personal information and records of the patients were de-identified before analysis to maintain patients’ anonymity. The protocol of this study was approved by the Ethics Institutional Review Board of Chang Gung Memorial Hospital (201600983B0).

### Patient enrollment and exclusion criteria

**Fig. 1:**
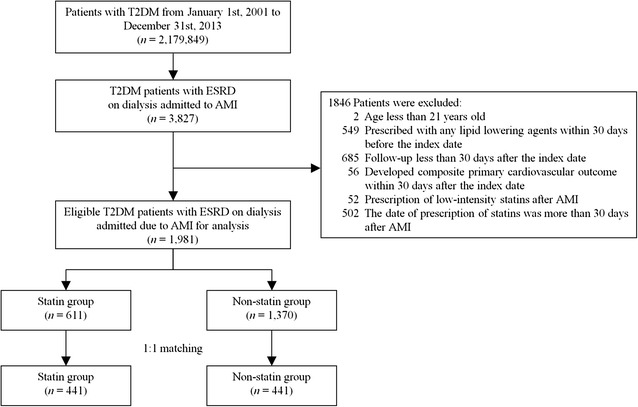
Flow chart of study subjects selection

This simulated prospective cohort study derived data from the NHIRD. Between January 1st, 2001 and December 31st, 2013, a total of 2,179,849 T2DM (ICD-9-CM: 250) patients were initially enrolled and, after applying exclusion criteria, a final total of 882 T2DM patients on dialysis who were hospitalized for AMI (ICD-9-CM: 410) were included in our study (Fig. 1). In addition to identifying T2DM patients using ICD-9-CM codes, we defined T2DM patients with at least 90 days of prescribed oral hypoglycemic agents or insulin injection within 1 year of the index hospitalization. The patients with ESRD receiving dialysis were identified based on a catastrophic illness certificate of ESRD that specifically defines those with a 24-h urine creatinine clearance rate of less than 5 ml/min who need long-term dialysis. In Taiwan, certificates of catastrophic illnesses are reviewed carefully by specialists on the committee of the Bureau of NHI because such illness may lead to overwhelming financial burdens and impoverishment. Therefore, in our study, the identification of patients with ESRD on dialysis is reliable and was proven valid in previous studies [25].

The index date was defined as the date on which a patient was admitted for AMI. The follow-up period was based upon the index date to date of death, loss of follow-up or until December 31st, 2013. All Patients’ baseline characteristics, comorbidities, prescribed medications and previous medical procedures, including percutaneous coronary intervention (PCI) and coronary artery bypass grafting (CABG), were identified. Patients were excluded if they met any of the following criteria: (a) age younger than 21 years; (b) use of any lipid-lowering agents within 30 days before the index date; (c) follow-up for less than 30 days after the index date; (d) major adverse cardiovascular events (defined as cardiovascular death, non-fatal myocardial infarction or non-fatal stroke) within 30 days of the index date; (e) prescription of low-intensity statins after AMI or (f) the date of statins prescription was more than 30 days after AMI. The exclusion criteria are shown in Fig. 1.

### Statins exposure

Moderate- (lowering LDL-C 30 to < 50%) or high-intensity (lowering LDL-C ≥ 50%) statins were classified according to the 2013 American College of Cardiology/American Heart Association guideline [11]. Study subjects were divided into a statin group receiving either moderate- or high-intensity statins after AMI and a non-statin group not receiving statins after AMI. The distributions and doses of statins in our study are shown in Additional file 1: Appendix S1.

### Outcomes and covariate measurements

Baseline comorbidities were identified by ICD-9-CM diagnosis codes and prescribed medications during hospitalization for AMI (Table 1). Primary outcomes were defined as composite events of cardiovascular death, non-fatal myocardial infarction and non-fatal ischemic stroke. The secondary composite outcomes included all-cause mortality, hospitalization for heart failure and hemorrhagic stroke. Safety outcomes were defined as acute hepatitis, rhabdomyolysis, newly diagnosed dementia and newly diagnosed malignant neoplasm during the follow-up period. Similarly, acute hepatitis, rhabdomyolysis, newly diagnosed dementia and newly diagnosed malignant neoplasms were identified using ICD-9-CM codes [25–27]. Cardiovascular death, death, and causes of death were defined as in the registry data of NHIRD [27].

**Table 1:** Characteristics of the study patients before and after propensity score matching

Variable	Before matching			After matching		
	Statin (*n* = 611)	Non-statin (*n* = 1370)	*P* value	Statin (*n* = 441)	Non-statin (*n* = 441)	*P* value
Age (year)	64.6 ± 10.2	67.5 ± 10.1	<.001	65.8 ± 10.2	65.6 ± 10.1	.844
Age group (years)			<.001			.933
21–60	210 (34.4)	324 (23.6)		131 (29.7)	136 (30.8)	
61–80	365 (59.7)	891 (65.0)		276 (62.6)	271 (61.5)	
> 80	36 (5.9)	155 (11.3)		34 (7.7)	34 (7.7)	
Gender			.038			.636
Male	323 (52.9)	793 (57.9)		236 (53.5)	243 (55.1)	
Female	288 (47.1)	577 (42.1)		205 (46.5)	198 (44.9)	
Dialysis			.033			1.000
Hemodialysis	558 (91.3)	1287 (93.9)		402 (91.2)	402 (91.2)	
Peritoneal dialysis	53 (8.7)	83 (6.1)		39 (8.8)	39 (8.8)	
Dialysis duration (year)	3.1 ± 2.9	3.1 ± 3.1	.949	3.3 ± 3.0	3.3 ± 3.3	.860
Diabetes mellitus duration (year)	11.3 ± 3.4	10.1 ± 3.8	<.001	11.2 ± 3.3	11.3 ± 3.4	.563
Comorbidity						
Hypertension	512 (83.8)	1045 (76.3)	<.001	367 (83.2)	363 (82.3)	.721
Dyslipidemia	428 (70.0)	412 (30.1)	<.001	259 (58.7)	254 (57.6)	.733
Heart failure	347 (56.8)	640 (46.7)	<.001	231 (52.4)	230 (52.2)	.946
Old myocardial infarction	209 (34.2)	505 (36.9)	.256	156 (35.4)	141 (32.0)	.285
Atrial fibrillation	49 (8.0)	113 (8.2)	.864	35 (7.9)	33 (7.5)	.801
Peripheral arterial disease	99 (16.2)	228 (16.6)	.808	69 (15.6)	70 (15.9)	.926
Chronic obstructive pulmonary disease	42 (6.9)	110 (8.0)	.372	30 (6.8)	29 (6.6)	.893
Malignancy	45 (7.4)	107 (7.8)	.731	33 (7.5)	34 (7.7)	.899
Cirrhosis	6 (1.0)	46 (3.4)	.002	6 (1.4)	6 (1.4)	1.000
Gout	43 (7.0)	90 (6.6)	.700	28 (6.3)	33 (7.5)	.507
Previous PCI	201 (32.9)	385 (28.1)	.031	143 (32.4)	136 (30.8)	.612
Previous CABG	49 (8.0)	97 (7.1)	.460	33 (7.5)	32 (7.3)	.897
Old ischemic stroke	172 (28.2)	420 (30.7)	.260	124 (28.1)	130 (29.5)	.655
Old hemorrhage stroke	16 (2.6)	33 (2.4)	.781	12 (2.7)	10 (2.3)	.666
History of bleeding (major bleeding)	308 (50.4)	693 (50.6)	.943	227 (51.5)	223 (50.6)	.788
Medication						
Aspirin	429 (70.2)	648 (47.3)	<.001	287 (65.1)	291 (66.0)	.777
Clopidogrel	494 (80.9)	670 (48.9)	<.001	338 (76.6)	331 (75.1)	.582
Warfarin	12 (2.0)	15 (1.1)	.123	11 (2.5)	9 (2.0)	.651
ACEI/ARB	343 (56.1)	481 (35.1)	<.001	220 (49.9)	226 (51.2)	.686
β-blocker	370 (60.6)	487 (35.5)	<.001	239 (54.2)	225 (51.0)	.345
Sulfonylurea	122 (20.0)	189 (13.8)	<.001	91 (20.6)	86 (19.5)	.674
Thiazolidinediones	13 (2.1)	38 (2.8)	.402	12 (2.7)	11 (2.5)	.833
Insulin	276 (45.2)	472 (34.5)	<.001	185 (42.0)	177 (40.1)	.584

*ACEi/ARB* angiotensin-converting-enzyme inhibitor/angiotensin receptor blockers, *CABG* coronary artery bypass grafting, *PCI* percutaneous coronary intervention

### Statistical analyses

The comparison cohort was matched with the statin group by a 1:1 ratio in terms of patient’s characteristics, baseline comorbidities, prescribed non-study medications (listed in Table 1), and index year and month using propensity score matching (PSM) to minimize potential selection bias and to simulate a prospective cohort study. Clinical characteristics between these two study groups were compared by Chi square test for categorical variables and by independent sample t test for continuous variables. Differences between the two study groups in time of the first occurrence of a predefined primary or secondary outcome after index date were determined by Cox proportional hazard models in which the study group (statin group versus non-statin group) was the only explanatory variable. Time-to-event outcomes were analyzed by predefined periods, including 3, 6 months, 1 year and until the final follow-up for each study group using the Kaplan–Meier method and log-rank test. A *P* value of less than .05 was considered statistically significant. All data analyses were performed using the SAS version 9.4 (SAS Institute, Cary, NC).

## Results

### Study population

A total of 2,179,849 T2DM patients were initially enrolled between January 1st, 2001 and December 31st, 2013, among whom 3827 T2DM patients on dialysis were admitted for AMI. After applying the exclusion criteria, a total of 1981 T2DM patients with ESRD on dialysis who were hospitalized for AMI were eligible for our study cohort. After PSM was used to reduce potential confounding and selection bias, the data of 882 patients were finally included for analysis (Fig. 1).

### Baseline characteristics

Among the 882 included patients, 441 (50%) were in the statin group and 441 matched patients (50%) were in the non-statin group. The mean age for the overall cohort was 65.7 years (standard deviation [SD] = 10.2 years). The mean follow-up period was 1.7 years (SD = 1.7 years) and the maximum follow-up time was 8 years. After PSM, the two study groups were well matched in terms of baseline characteristics, comorbidities and non-study medications (Table 1). More than 90% of the patients received hemodialysis with a mean duration of 3.3 years (SD = 3.2 years). The most common co-morbidity was hypertension (83.2% vs. 82.3%), followed by dyslipidemia (58.7% vs. 57.6%) and heart failure (52.4% vs. 52.2%) in the statin and non-statin groups, respectively. In addition, patients with old myocardial infarction and old ischemic stroke in the statin group were 35.4 and 28.1%, respectively; in the non-statin group, those with old myocardial infarction and old ischemic stroke were 32.0 and 29.5%, respectively (Table 1).

### Primary outcomes

**Fig. 2:**
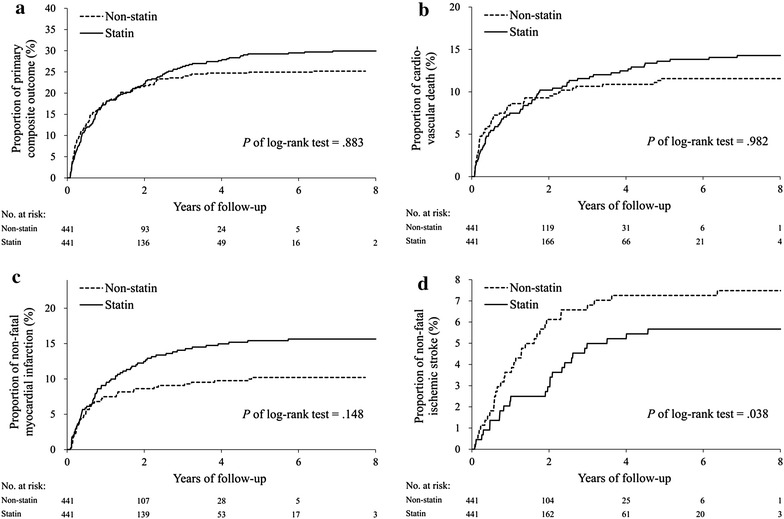
Cumulative probability of event rates in each study group for **a** primary composite outcome, **b** cardiovascular death, **c** non-fatal myocardial infarction, **d** non-fatal ischemic stroke. The primary composite outcome included cardiovascular death, non-fatal myocardial infarction and non-fatal ischemic stroke. The Kaplan–Meier event rate for the primary composite outcome at 8 years was 30.2% in the statin group compared with 25.2% in the non-statin group (HR, .98; 95% CI .76–1.27), but the risk of non-fatal ischemic stroke was lower in the statin group (5.9%) than that in the non-statin group (7.5%) (HR, .58; 95% CI .35–.98)

**Fig. 3:**
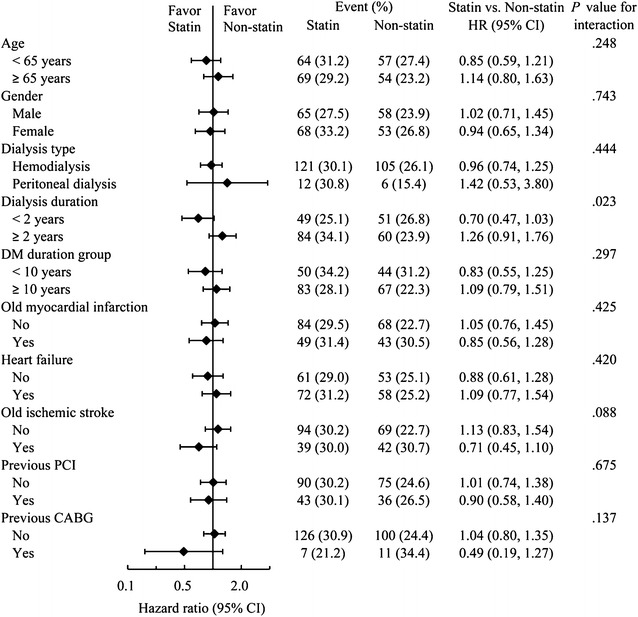
Subgroup analyses for the primary composite outcome at final follow-up

Events of primary composite outcomes occurred in 133 patients (30.2%) in the statin group and in 111 patients (25.2%) in the control group (HR, .98; 95% CI .76–1.27) at final follow-up (Table 2). With regard to the individual composite outcome, there was a significant difference in the risk of non-fatal ischemic stroke (HR, .58; 95% CI .35–.98) which favored the statin users. The cumulative incidence of the primary composite outcome and each component in the two study groups were plotted (Fig. 2a–d). In subgroup analysis, the effects of statin therapy suggested the primary composite outcome was better in patients who received dialysis for less than 2-years duration (*P* for interaction = .023), although the total effect was neutral (Fig. 3).

**Table 2:** Event numbers and hazard ratio of the primary outcome between the study cohorts

Outcome	Number of event (%)		Statin vs. non-statin	
	Statin (*n* = 441)	Non-statin (*n* = 441)	HR (95% CI)	*P* value
3 Month follow-up				
Non-fatal ischemic stroke	2 (.5)	5 (1.1)	.40 (.08, 2.05)	.271
Non-fatal myocardial infarction	13 (2.9)	12 (2.7)	1.09 (.50, 2.38)	.834
Cardiovascular death	13 (2.9)	21 (4.8)	.62 (.31, 1.24)	.173
Primary composite outcome^a^	27 (6.1)	37 (8.4)	.73 (.44, 1.20)	.211
6 Month follow-up				
Non-fatal ischemic stroke	6 (1.4)	8 (1.8)	.73 (.25, 2.10)	.559
Non-fatal myocardial infarction	26 (5.9)	24 (5.4)	1.06 (.61, 1.85)	.835
Cardiovascular death	24 (5.4)	27 (6.1)	.87 (.51, 1.52)	.633
Primary composite outcome^a^	52 (11.8)	56 (12.7)	.91 (.62, 1.32)	.610
1 year follow-up				
Non-fatal ischemic stroke	10 (2.3)	16 (3.6)	.58 (.26, 1.27)	.170
Non-fatal myocardial infarction	40 (9.1)	33 (7.5)	1.16 (.73, 1.83)	.541
Cardiovascular death	33 (7.5)	37 (8.4)	.85 (.53, 1.36)	.506
Primary composite outcome^a^	77 (17.5)	78 (17.7)	.93 (.68, 1.28)	.660
At the end of follow-up				
Non-fatal ischemic stroke	26 (5.9)	33 (7.5)	.58 (.35, .98)	.040
Non-fatal myocardial infarction	69 (15.6)	45 (10.2)	1.32 (.91, 1.92)	.149
Cardiovascular death	63 (14.3)	51 (11.6)	1.00 (.69, 1.45)	.982
Primary composite outcome^a^	133 (30.2)	111 (25.2)	.98 (.76, 1.27)	.883

*CI* confidence interval, *HR* hazard ratio

^a^Anyone of cardiovascular death, non-fatal myocardial infarction, non-fatal ischemic stroke

### Secondary outcomes

For secondary outcomes, patients treated with statins had a lower risk of all-cause mortality (HR, .70; 95% CI .59–.84) compared to those in the non-statin group (Table 3). In addition, no significant differences were found between the statin group and non-statin group in the respective incidence of hemorrhagic stroke (.9 and .5%; *P* = .810), hospitalization for heart failure (13.4 and 10.0%; *P* = .714), acute hepatitis (.2 and .7%; *P* = .194), rhabdomyolysis (.9 and .2%; *P* = .279), newly diagnosed dementia (0 and .2%; *P* = not applicable) or newly diagnosed malignancy (3.2 and 2.0%; *P* = .844) (Table 3).

**Table 3:** Secondary outcomes at final follow-up

Outcome	Number of event (%)		Statin vs. non-statin	
	Statin (*n* = 441)	Non-statin (*n* = 441)	HR (95% CI)	*P* value
All-cause mortality	250 (56.7)	271 (61.5)	.70 (.59, .84)	<.001
Hemorrhage stroke	4 (.9)	2 (.5)	1.23 (.22, 6.84)	.810
Heart failure	59 (13.4)	44 (10.0)	1.08 (.73, 1.60)	.714
Acute hepatitis	1 (.2)	3 (.7)	.22 (.02, 2.16)	.194
Rhabdomyolysis	4 (.9)	1 (.2)	3.36 (.38, 30.06)	.279
Newly diagnosed dementia	0 (.0)	1 (.2)	NA	NA
Newly diagnosed malignancy	14 (3.2)	9 (2.0)	1.09 (.46, 2.56)	.844

*CI* confidence interval, *HR* hazard ratio, *NA* not applicable

## Discussion

Although AMI is a life-threatening disease and statins reduce the incidence of cardiovascular events, evidence of the effectiveness of moderate- to high-intensity statin therapy in T2DM patients on dialysis after AMI is still limited [11, 28]. The strength of the present study is that it is the first nationwide, population-based study to evaluate the clinical outcomes of moderate- to high-intensity statins in T2DM patients on dialysis after a recent AMI. The results of our study suggest that the use of moderate- to high-intensity statins has a neutral effect on composite cardiovascular events but may reduce the risks of non-fatal ischemic stroke and all-cause mortality in this special population, without increasing the incidence of major complications such as hemorrhagic stroke, acute hepatitis, rhabdomyolysis or newly diagnosed dementia. The use of moderate- to high-intensity statins reduced the risk of non-fatal ischemic stroke by 42% and all-cause mortality by 30% during the mean follow-up of 1.7 ± 1.7 years. For non-fatal ischemic stroke and all-cause mortality, the numbers needed to treat were 62.5 and 20.8, respectively.

Our results for combined cardiac events reduction are compatible with those of previous randomized controlled trials (i.e., 4-D [7], AURORA [16] and SHARP [17] trials), which indicated that statins or statins combined with ezetimibe provided no significant benefits for patients on dialysis. This may be explained by the significant structural changes in the myocardium with functional abnormalities, a different pathology of vascular stiffness with calcification and propensity to arrhythmia attributed to sympathetic overactivity at end-stage renal disease [29–33]. Subgroup analysis in our study suggested that primary composite outcomes may be better in patients who received dialysis for less than 2-years duration; this finding is also correlated with the comment from the 4-D study that said the initiation of statins in T2DM patients on dialysis who already have ESRD (the average duration of dialysis is over 8 years) may come too late to translate into consistent improvement of cardiovascular outcomes [7]. According to the literature, in older women with diabetes on peritoneal dialysis, there might be an excess cardiovascular mortality [34]. Nevertheless, the primary composite outcome in subgroup analysis of the effect with moderate- to high-intensity statins showed no significant difference between hemodialysis and peritoneal dialysis.

Compared to the results of the 4-D study, which showed a significant increased risk of fatal stroke (HR 2.03, 95% CI 1.05–3.93) in the atovastatin group, and the AURORA study, which showed a neutral effect in non-fatal ischemic stroke with rosuvastatin, in our study, the statin group demonstrated significant risk reduction for non-fatal ischemic stroke (HR, .58; 95% CI .35–.98) at final follow-up. The actual reason for this discrepancy in findings is unclear. However, any of the following explanations may apply. First, the patients in our study were mostly an Asian population. By comparison, the 4D study was conducted in Germany and the AURORA study only enrolled 5% Asian patients. Because intracranial atherosclerosis is relatively common in Asia [35], the clinical significance of statin therapy could be different in Asian subjects compared to Western subjects. Second, the etiology of ischemic stroke is heterogeneous with large vessel disease, small vessel disease and embolism, and statins for stroke prevention may act differently according to different etiologies. Third, in our study, more patients had old ischemic stroke (28.1% in the statin group and 29.5% in the non-statin group) than did patients with old stroke or history of transient ischemic attack in the 4-D study (17.4% in the atovastatin group and 18.2% in the placebo group).

Our results for decreased all-cause mortality were compatible with the findings from a large prospective cohort of incident dialysis patients from the United States renal data system dialysis morbidity and mortality study wave 2 (USRDS DMMS-2) and the dialysis outcomes and practice patterns study, which both showed that statin use was associated with at least a 30% reduction in all-cause mortality [36, 37]. Although the precise mechanism of decreased all-cause mortality without reduction of CV death in our statin group was uncertain, the possible explanation could be the pleiotropic effects of statins, including lowering oxidative stress, improving endothelial dysfunction, reducing endothelial cell apoptosis, decreasing inflammation and beneficial to the immune system, regardless of the level of LDL-C [38, 39]. Patients on dialysis have increased levels of oxidative stress that could have an influence on non-cardiac functions and immune responses to infection [40]. The anti-oxidant properties of statins could possibly produce beneficial changes in non-cardiac functions and result in improved non-cardiac survival [36]. Therefore, the use of statins associated with reducing all-cause mortality was observed in many other special populations, such as patients with sepsis [41], cancer [42] or chronic obstructive pulmonary disease [43].

In the our study, the all-cause mortality (56.7% in the statin group and 61.5% in the non-statin group) was higher than that in the 4D and AURORA studies. There are several explanations for these discrepant findings. First, compared with other studies in which patients with a recent acute coronary syndrome were excluded, our study examined T2DM patients with ESRD on dialysis who had a recent AMI, making our study cohort at a much higher cardiovascular risk than that found in other studies. AMI is a catastrophic event in dialysis patients and more than 50% of all patients die within the first year [44, 45]. Second, the 4D [7] and AURORA [16] studies enrolled fewer patients with old myocardial infarction than those enrolled in our study (17.9% in the atorvastatin group and 17.3% in the placebo group; 10.5% in the rosuvastatin group and 9.8% in the placebo group). As a result, the patients in our study were exceptionally vulnerable to the complications of cardiovascular disease.

Regarding hemorrhagic stroke, a higher incidence of hemorrhagic stroke (HR 5.21, 95% CI 1.17–23.27) was observed in the rosuvastatin group of the AURORA study [16]. In contrast, in our study, no significant differences were found in the risk of hemorrhagic stroke (.9 and .5%; *P* = .810), although the number of events in the statin group was still higher than those in the non-statin group (4 vs. 2, respectively). Also, no significant differences were found in the other safety outcomes such as acute hepatitis, rhabdomyolysis, newly diagnosed dementia or newly diagnosed malignancy between the statin and non-statin groups.

Our study has several limitations. First, certain patient data, including smoking, lifestyle factors, body mass index, family history of cardiovascular disease, or laboratory parameters such as glycated hemoglobin levels, were not available. Nevertheless, we were able to include a wide range of variables related to outcomes, including duration of T2DM, major comorbidities and non-study medications, to make our two study groups well matched. Second, we assumed that patients had properly adhered to their treatment medications as reported in the claims data. Third, this is an observational trial and cause/effect relationships must be carefully interpreted. Furthermore, it remains unclear whether the findings of our study are applicable to other ethnicities. Despite these limitations, our real-world nationwide, population-based data is still of value to help fill the gap of evidence and answer uncertain questions, because randomized controlled trials are not always feasible due to considerations of cost, ethical, or time.

## Conclusions

In patients with T2DM on dialysis after AMI, the use of moderate- to high-intensity statins has a neutral effect on composite cardiovascular events but may reduce risks of non-fatal ischemic stroke and all-cause mortality without increasing the incidence of major complications such as acute hepatitis, rhabdomyolysis, hemorrhagic stroke, newly diagnosed dementia or newly diagnosed malignancy in this special population.

## Additional file

**Additional file 1.** The distributions and doses of moderate- to high-intensity statins according to the 2013 American College of Cardiology/American Heart Association guideline.

## Abbreviations

T2DM: type 2 diabetes mellitus
AMI: acute myocardial infarction
CKD: chronic kidney disease
CVD: cardiovascular disease
ESRD: end-stage renal disease
LDL-C: low-density lipoprotein cholesterol
HMG-CoA: 3-hydroxy-3-methylglutaryl coenzyme A
ASCVD: atherosclerotic cardiovascular disease
AMI: acute myocardial infarction
