# Supplementary material for: Moderate- to high-intensity statins for secondary prevention in patients with type 2 diabetes mellitus on dialysis after acute myocardial infarction
Source: Diabetol Metab Syndr. 2017 Sep 19;9:71. doi: 10.1186/s13098-017-0272-7 (PMC5605978; doi:10.1186/s13098-017-0272-7)
Supplement: Supplementary file 1 — Additional file 1. The distributions and doses of moderate- to high-intensity statins according to the 2013 American College of Cardiology/American Heart Association guideline. [file 13098_2017_272_MOESM1_ESM.docx]

**Appendix S1.** The distributions and doses of moderate- to high-intensity statins according to the 2013 American College of Cardiology/American Heart Association guideline

|  | Before matching  (*n* = 611) | After matching  (*n* = 441) |
| --- | --- | --- |
| Intensity |  |  |
| High | 110 (18.0) | 81 (18.4) |
| Moderate | 501 (82.0) | 360 (81.6) |
| Brand and dose |  |  |
| Atorvastatin 40-80 mg^†^ | 110 (18.0) | 81 (18.4) |
| Rosuvastatin 20-40 mg^†^ | 0 (0.0) | 0 (0.0) |
| Atorvastatin 10-20 mg^‡^ | 248 (40.6) | 175 (39.7) |
| Rosuvastatin 5-10 mg^‡^ | 166 (27.2) | 116 (26.3) |
| Simvastatin 20-40 mg^‡^ | 29 (4.7) | 27 (6.1) |
| Pravastatin 40-80 mg^‡^ | 7 (1.2) | 5 (1.1) |
| Lovastatin 40 mg^‡^ | 0 (0.0) | 0 (0.0) |
| Fluvastatin 80 mg^‡^ | 46 (7.5) | 34 (7.7) |
| Pitavastatin 2-4 mg^‡^ | 5 (0.8) | 3 (0.7) |

^†^high-intensity statins; ^‡^moderate-intensity statins
